# Influence of Defects and Microstructure on the Thermal Expansion Behavior and the Mechanical Properties of Additively Manufactured Fe-36Ni

**DOI:** 10.3390/ma17174313

**Published:** 2024-08-30

**Authors:** Moritz Kahlert, Thomas Wegener, Leonard Laabs, Malte Vollmer, Thomas Niendorf

**Affiliations:** Institute of Materials Engineering-Metallic Materials, University of Kassel, Moenchebergstr. 3, 34125 Kassel, Germany; kahlert@uni-kassel.de (M.K.); l.laabs@uni-kassel.de (L.L.); vollmer@uni-kassel.de (M.V.); niendorf@uni-kassel.de (T.N.)

**Keywords:** additive manufacturing (AM), selective laser melting (SLM), Invar 36 (Fe_64_Ni_36_; Fe-36Ni), microstructure, mechanical properties, thermal expansion, design of experiment (DOE)

## Abstract

Laser-based powder bed fusion of metals (PBF-LB/M) is a widely used additive manufacturing process characterized by a high degree of design freedom. As a result, near fully dense complex components can be produced in near-net shape by PBF-LB/M. Recently, the PBF-LB/M process was found to be a promising candidate to overcome challenges related to conventional machining of the Fe_64_Ni_36_ Invar alloy being well known for a low coefficient of thermal expansion (CTE). In this context, a correlation between process-induced porosity and the CTE was presumed in several studies. Therefore, the present study investigates whether the unique thermal properties of the PBF-LB/M-processed Fe_64_Ni_36_ Invar alloy can be tailored by the selective integration of defects. For this purpose, a full-factorial experimental design, representing by far the largest processing window in the literature, was considered, correlating the thermal expansion properties with porosity and hardness. Furthermore, the microstructure and mechanical properties were investigated by scanning electron microscopy and quasi-static tensile tests. Results by means of statistical analysis reveal that a systematic correlation between porosity and CTE properties could not be determined. However, by using specific process parameter combinations, the microstructure changed from a fine-grained fan-like structure to a coarse columnar structure.

## 1. Introduction

The single γ-phase Fe-36Ni Invar alloy is known for its low thermal expansion up to the Curie temperature (T_C_) and is, therefore, widely used for high-precision industrial applications featuring superior dimensional stability requirements [1,2]. The unique property of an invariance to thermal expansion is based on a physical effect, i.e., the so-called spontaneous volume magnetostriction. Accordingly, with an increase in temperature, the repulsive magnetic properties decrease in the same order of magnitude as the lattice expansion [3]. Above T_C_, the magnetic properties disappear, which leads to a temperature expansion similar to conventional materials. This effect was first described by Guillaume in 1898 [1]. Components made of Invar are conventionally produced by turning, milling, rolling, or electroplating [4]. However, this is challenging due to the high ductility and low thermal conductivity and, therefore, causes high tool wear and, thus, high production costs, especially for complex parts often required for high-precision applications [5,6,7,8].

Laser-based powder bed fusion of metals (PBF-LB/M) is highly qualified for the production of complex parts and, therefore, also a promising candidate to overcome the challenges related to conventional processing of Invar. The manufacturing process enables the creation of complex free-form geometries directly from a 3D-model without the use of tools [9,10,11,12]. Components manufactured by PBF-LB/M are characterized by high density, which, in most cases, is close to conventionally manufactured materials. The PBF-LB/M process is a layer-by-layer additive manufacturing (AM) technique in which a fine powder layer of usually 30–100 µm is applied and melted in a repetitive process using a laser [13,14]. Typical applications are implants for the biomedical sector as well as complex lightweight structures for aerospace [13,15].

Generally, the processability of dense Invar components manufactured by PBF-LB/M has already successfully been shown in several studies [6,7,16,17,18,19,20]. For a comprehensive overview of microstructure evolution, as well as mechanical properties of PBF-LB/M processed Invar alloy, the reader is referred to [16] at this point. It has been found that different process parameter combinations lead to different densities and defect types. For example, Asgari et al. [7] found an increase in density with increasing bulk energy in the range from 119 J/mm^3^ to 190 J/mm^3^. This is in contrast to the investigations by Yakout et al. [6], reporting on the formation of keyhole defects above a laser energy density range of 60–75 J/mm^3^. According to the authors, this range represents the optimal process window. In another study of these authors [20], a critical energy density of 86.8 J/mm^3^, leading to maximum densities of the manufactured parts, was highlighted. Furthermore, below a volume energy of 41.7 J/mm^3^, lack of fusion defects were found due to a lack of bonding of the layers [17].

The resulting microstructure of PBF-LB/M Invar components is strongly influenced by the volume energy and laser power applied. According to Zhu et al. [21], heat accumulation from higher laser powers leads to columnar grains, which grow beyond the melt pool and can reach dimensions of several millimeters in length. Lower laser powers lead to a less pronounced temperature gradient and, thus, to a finer microstructure mostly being in the range of the dimension of the melt pool [21]. In good agreement, different authors [7,17,21,22] reported on austenitic microstructures in the PBF-LB/M-processed Invar specimens, which primarily exhibit a grain orientation in [100]-direction. In contrast, Qui et al. [22] were also able to detect fine α-precipitates featuring a size of approximately 100 nm.

Concerning the mechanical properties, Wegener et al. [16] demonstrated a homogeneous distribution of the hardness of 158 HV1 and a ductile deformation behavior under monotonic tensile loading with an elongation at break of 43–47% and a concomitant ultimate tensile strength (UTS) of 445 MPa. Yakout et al. [20] reported a brittle-to-ductile transition energy density (E_C_ = 52.1 J/mm^3^). Below this value, the parts were characterized by void formation, low density and brittle fracture, whereas higher energy density values led to the vaporization of some alloying elements. Accordingly, the relative density of the specimen affected the mechanical properties of the built parts. Before reaching E_C,_ the yield strength (YS), UTS, and elongation increased with increasing energy density. Above E_C,_ an increase in the energy density resulted in a decrease in YS and UTS, accompanied by an increase in ductility. PBF-LB/M Invar specimens processed with E_C_ = 52.1 J/mm^3^ were characterized by YS and UTS and elongation values of approximately 355 MPa, 450 MPa, and 50%, respectively. Consistently, different authors [17,22,23] reported UTS values in the range of 400–530 MPa depending on the relative density of the specimens.

Since the functional properties of Invar are paramount for almost all envisaged applications, the thermal expansion properties and, thus, the effectiveness of the Invar effect after PBF-LB/M processing are of particular relevance. Harrison et al. [23] revealed a lower thermal expansion compared to conventionally manufactured Invar, featuring a value of −0.355 × 10^−6^ 1/K in the range of 30 °C to 100 °C and 0.889 × 10^−6^ 1/K in the range of 100 °C up to 200 °C [23]. Wegener et al. [16] reported a similar thermal expansion of approximately 1.8 × 10^−6^ 1/K. In addition, Asgari et al. [7] were able to identify a correlation between density and thermal expansion in their studies. In accordance, the coefficient of thermal expansion (CTE) increased with increasing relative density of the specimen. In contrast, Yakout et al. [17] reported on specimens that exhibit a low CTE built with both, a very low and very high volume energy. Lower CTE values, as compared to conventionally processed Invar counterparts, were generally rationalized by the process-induced porosity being present in the PBF-LB/M Invar components.

As the introduction clearly outlines, a reasonable knowledge base including the influence of different process parameters on the resulting microstructure and the mechanical properties under tensile loading has already been comprehensively elaborated for PBF-LB/M Invar. However, several studies presume a correlation between process-induced porosity and thermal expansion properties. In order to clarify this relationship, a full-factorial design of experiment (DOE), representing by far the largest processing window in the literature, was established in the present study, which includes both, high-density and low-density specimens in a wide range of parameters. These specimens were analyzed by means of their thermal expansion behavior using a dilatometer, and the results were linked to the density obtained by optical microscopy (OM). Moreover, the data obtained from the DOE were analyzed using evaluation methods taking into account statistics. In addition, the microstructure of several specimens was investigated using electron backscatter diffraction (EBSD) and the mechanical properties were characterized by hardness measurements and tensile tests. From the results shown, correlations between thermal expansion behavior and microstructural as well as mechanical properties can be drawn.

## 2. Materials and Methods

For manufacturing of the specimens investigated in the present study, a SLM280HL PBF-LB/M system from SLM Solutions GmbH (Lübeck, Germany) was used. The system is equipped with a dual-laser system consisting of a 400 W laser with a Gaussian profile and a 1000 W laser with a top-hat profile, respectively. However, for better comparability with previous work, only the laser with a maximum power of 400 W was used. For the investigation presented, Fe-36Ni powder obtained from SLM Solutions GmbH was used. According to particle size distribution analysis provided by the powder supplier, the mean diameter, as well as values of d_10_, d_50_, and d_90_, were determined as 36 µm, 18 µm, 33 µm, and 58 μm, respectively. For manufacturing of specimens, a DOE (Supplementary Data Table S1) was established. Within this DOE, the following PBF-LB/M process parameters were varied: laser power (100 W, 200 W, 300 W), scan speed (700 mm/s, 800 mm/s, 900 mm/s, 1000 mm/s), hatch distance (0.05 mm, 0.1 mm, 0.15 mm), and layer thickness (0.05 mm, 0.1 mm). In addition to preliminary work conducted by some of the authors [16], the selection of the parameter ranges of the DOE was primarily based on studies reported by Yakout et al. [17,20]. In these studies, the authors also analyzed a full factorial experimental design with 27 specimens in total and reported on a correlation between process-induced defects and CTE behavior. The approach presented in the present study results in a full factorial study of 72 parameter combinations covering a volume energy range from 6.667 J/mm^3^ to 171.429 J/mm^3^. As a common feature, a bidirectional scanning strategy with 90° rotation between layers was chosen in order to reduce residual stresses and to avoid the accumulation of process-induced defects in the course of successive layers, such as insufficient melting at reversal points of the scan vectors. For improved vapor removal in the PBF-LB/M process, the specimens were manufactured with an angle of 45° to the gas flow direction. Moreover, the PBF-LB/M process was conducted under an argon inert gas atmosphere and at a 200 °C substrate plate temperature. A gas flow velocity of 20 m/s was chosen to ensure adequate smoke removal. Using the process parameters described, four cylindrical specimens with a diameter of 4 mm and a length of 25 mm were fabricated for each process parameter combination in order to analyze the density, thermal expansion properties, and hardness. Density was determined optically based on the black and white ratio method using ImageJ (Version 1.54g) [24]. Here, a micrograph of a cross-section of an entire specimen, perpendicular to the build direction, was used as the area of interest. This optical micrograph was taken using a Keyence VHX7000 microscope (Osaka, Japan). A Dil802 dilatometer from Bähr–Thermoanalyse GmbH (Hüllhorst, Germany) was used to analyze the thermal expansion behavior within a temperature range of 50–300 °C. Here, the cylindrical specimens featuring a length of 25 mm and a diameter of 4 mm were analyzed in as-built condition (with respect to their surface state). A heating rate of 10 K/min was selected and the investigation was performed under an argon flow of 10 l/min. A Zeiss ULTRA GEMINI scanning electron microscope (SEM) from Zeiss GmbH (Oberkochen, Germany) equipped with a QUANTAX electron backscatter diffraction (EBSD) unit from Bruker Corporation (Billerica, MA, USA) was used to examine the microstructure. For EBSD measurements, the specimens were mechanically ground down to 8.4 µm grit size (P2500 according to the FEPA P norm) using silicon carbide (SiC) paper and then vibropolished for 18 h using the final polishing agent OPS Non-dry (Struers, Ballerup, Denmark). All measurements were performed at an accelerating voltage of 20 kV, using the same magnification, aperture size, and step size, i.e., 100×, 120 µm, and 1.48 µm. Atex software (Version 4.14) [25] was used for post-processing of the EBSD data. For the HV1 hardness measurements, a V100 hardness tester from Leco Corporation (St. Joseph, MI, USA) was used in accordance with DIN ISO 6507 [26], employing a load of 9.81 N. In each case, a mean value was calculated from three individual measurements.

Based on the results obtained by the investigations described above, six parameter combinations featuring a wide range of porosity, all being characterized by low thermal expansion, were selected (see Section 3 for details) in order to determine the mechanical properties by means of tensile tests in the as-built condition, i.e., without any post-processing such as heat treatment or surface finishing. The surface roughness of the six parameter sets in as-built condition was analyzed by optical focus-variation using the Keyence VHX7000 microscope described above. A magnification of 500× was used to analyze a length of 3 mm with focal steps of 1 µm to ensure sufficient resolution. For each parameter set two different areas of a specimen were probed, and two lines parallel to the build/load direction were analyzed. Following this procedure, a mean roughness value was determined for each of the six selected parameter combinations. Uniaxial room temperature tensile tests were performed on a servo-hydraulic testing machine. For this purpose, the specimen geometry presented by Oevermann et al. [27] was adapted to the dilatometer specimens with 4 mm diameter in the gauge length (15 mm) to avoid size differences (cf. Figure 4b). The tensile tests were performed under displacement control with a constant crosshead speed of 2 mm/min. The strains were recorded using an extensometer with a maximum strain capability of 50% (Sandner Messtechnik GmbH, Biebesheim am Rhein, Germany). In total, three tensile tests were performed for each condition considered. To analyze the fracture surface, a CamScan MV 2300 SEM (Tescan Group, a.s., Brno, Czech Republic) operating at 20 kV was used.

## 3. Results and Discussion

Density, hardness, and CTE measurements were conducted on all specimens considered in the DOE. The corresponding results are summarized in Table S2 of the Supplementary Data. Regarding the thermal expansion properties, mean CTE values are provided for two different temperature ranges, i.e., 50–100 °C, as well as 100–200 °C. Analyzing the correlation between the volume energies used and the resulting densities of the specimen as a first step, differences to the common literature on PBF-LB/M-processed Invar are revealed. In their study on the selection of process parameters in additive manufacturing for aerospace alloys, Yakout et al. [6] reported that the manufacturing of dense PBF-LB/M Invar components requires process parameter combinations, which are characterized by volume energy densities approximately between 60 and 75 J/mm^3^. In contrast, the investigations of the present study reveal that, e.g., the parameter combination with the number 70 featuring a volume energy of 30 J/mm^3^ is already characterized by a relative density of 98.51%. Comparing this density value in turn with a counterpart specimen built with an almost similar volume energy of 29 J/mm^3^, the latter parameter combination with the number 2 exhibits a relative density of only 71.17%. This observation clearly shows that a large number of parameters have an influence on the resulting relative density of a specimen manufactured by PBF-LB/M, and that the volume energy by itself can only provide a rough indicator point, however, is no guarantee for a dense specimen. This particularly holds true when different PBF-LB/M systems or powder suppliers are considered. Comparing the hardness values of the individual parameter combinations with their relative densities as a next step, it is noticeable that especially conditions characterized by low densities exhibit a very high deviation from average hardness values reported for as-build PBF-LB/M-processed Invar, e.g., 158 HV1 reported by Wegener et al. [16] for specimens featuring a relative density of 99.6%. For example, Specimen Number 12, with a particularly low relative density of 38.33%, features a hardness of only 50 HV. In contrast, Specimen 53 being characterized by a relative density of 99.66% reveals a hardness of 150 HV. These results can be attributed to the fact that if during hardness measurement the indenter is applied to an area of the specimen that is not strongly bonded to the surrounding material due to a high porosity, the resistance against indentation is very low, leading to low hardness values. As a result, no valid measurement can be conducted at that point.

### 3.1. Statistical Analysis of the DOE

**Figure 1:**
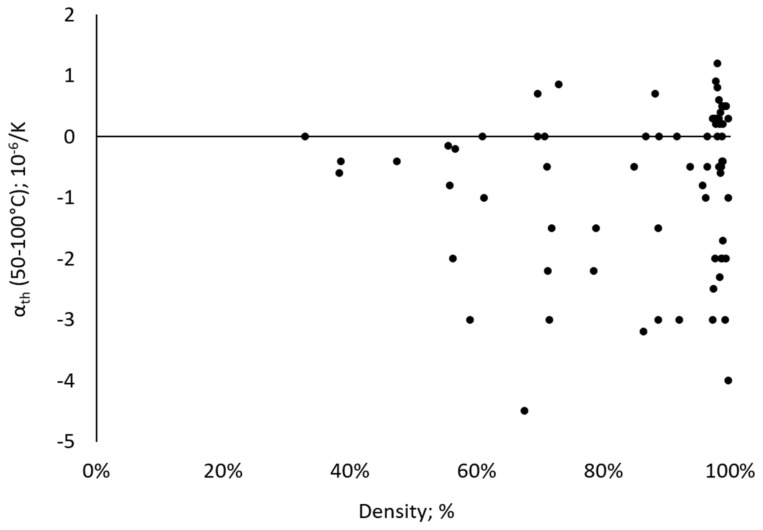
Mean thermal expansion coefficient (α_th_) in the temperature range from 50–100 °C plotted against the relative density of the individual specimen.

As detailed in the introduction section, Asgari et al. [7] reported on a reduction of the thermal expansion behavior due to an increase of the porosity. Moreover, Yakout et al. [17] rationalized lower CTE values of PBF-LB/M-processed Invar as compared to conventionally processed counterparts by voids, causing an increase in the magnetic dipole moment and, eventually, a reduction in the coefficient of thermal expansion. In order to analyze possible correlations between relative density and thermal expansion properties of PBF-LB/M-processed Invar, CTE values of the individual DOE specimens in the temperature range from 50–100 °C were plotted against the resulting relative density in Figure 1. Generally, the effectiveness of the Invar effect for the material analyzed in the present study can be derived, as the CTE of the PBF-LB/M-processed Invar within this work shows a behavior similar to the corresponding literature reporting on additively manufactured Invar. As a reference, Harrison et al. [23] observed a CTE of 3.56 × 10^−6^ 1/K in the thermal range from 30 °C to 279 °C and a CTE of −0.355 × 10^−6^ 1/K in the thermal range from 30 °C to 100 °C being similar to the values observed in the present work. Moreover, Li et al. [15] reported on similar thermal expansion properties of PBF-LB/M-processed Invar with a CTE of about 3.6 × 10^−6^ 1/K in the temperature range from 25 °C to 220 °C. However, from the results presented in Figure 1, it can be seen that both specimens with low relative density and specimens with high relative density are characterized by low CTE values, many of them even being negative in the temperature range considered. The correlation between density and CTE previously described in the literature, e.g., by Asgari et al. [7], could, therefore, not be confirmed from the results shown here. This is also underlined by analysis of the data using the statistical software Minitab^®^ (Version 19.2020.1) [28]. In this context, the Pearson correlation coefficient *r* can be used to examine the strength and direction of the linear relationship between two continuous variables. Accordingly, a Pearson correlation, representing the most common method for correlation, with a confidence interval (CI) of 95% for the correlation coefficient, was applied to the density and the CTE in the temperature range from 50–100 °C (CTE1), as well as to the density and the CTE in the temperature range from 100–200 °C (CTE2). The results are shown in the form of matrix plots in Figure S1 of the Supplementary Data. It becomes obvious that in both cases low *r*-values of 0.077 and 0.044 are obtained for CTE1 and CTE2, respectively. As a result, the lack of correlation discussed above is confirmed as a correlation close to zero indicates that there is no linear relationship between the variables. Moreover, both Pearson’s correlation coefficients reveal no statistical significance, as the statistical significance level, i.e., the *p*-values (0.523 and 0.711 for CTE1 and CTE2, respectively) do not satisfy the common cut-off for a statistical significance of *p* < 0.05. Thus, it can be concluded that the full-factorial experimental design of the present study, representing by far the largest processing window in the literature, refutes the assumptions of a correlation between density and CTE as previously described in literature. Accordingly, alternative factors influencing the CTE behavior such as microstructure (being assessed in a later section of the manuscript for selected parameter combinations) or residual stress (as e.g., assumed in the study of Harrison et al. [23] without experimental evidence) need to be considered and assessed in follow-up studies.

In order to further assess the possible influence of the different factors of the DOE, i.e., laser power, scan speed, hatch distance, and layer thickness, on the CTE behavior of the specimens, the full-factorial design was analyzed using Minitab^®^ by means of general factorial regression considering the CTE as a covariate. The results are shown in the form of Pareto charts in Figure S2 of the Supplementary Data. These Pareto plots allow to visually identify the important effects and compare the relative magnitude of the various effects. The red dashed line in the Pareto diagrams is used to evaluate the significance of factors in relation to the 95% statement probability (α = 0.05). Influence factors to the right of the red dashed line are significant. The length of the bars in comparison to each other indicates how high or low the significance is in relation to each other. As can be deduced, neither the individual factors nor the two-way or three-way interactions reveal significance in their impact on CTE1 and CTE2, respectively. Interestingly, as a common feature in both cases, the largest relative magnitude can be seen for the two-way interaction of the layer thickness and scan speed (indicated by BD). As a result, future studies should focus on further variation of these two factors in order to see whether a significance can be revealed or not.

### 3.2. Microstructure Characterization

**Figure 2:**
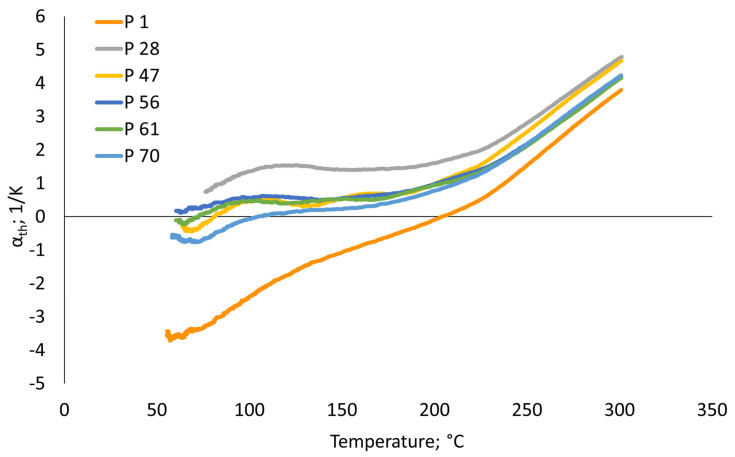
Thermal expansion coefficient (α_th_) of selected specimens from the DOE considered plotted against the temperature.

For further investigations including microstructural analysis by EBSD as well as tensile testing, six exemplary parameter sets were selected from all parameter combinations shown in Figure 1. Bold letters in Tables S1 and S2 of the Supplementary Data additionally mark these parameter sets. The selection was generally intended to provide a wide range of densities while capturing the parameters with the lowest CTEs at the same time, eventually in order to narrow down possible influences on the specific CTE behavior, which are not related to the density. Moreover, some selected conditions, i.e., Numbers 28 and 47, include parameter sets which showed different densities at the same volume energies. Reasoned by the largest deviations in the thermal expansion curve compared to all counterpart specimens, Parameter Combination 1 was finally also selected for an in-depth analysis. The thermal expansion behavior between 50 °C and 300 °C for the six selected parameter combinations is shown in Figure 2. The process parameters, as well as the resulting volume energy and the density of these selected parameter sets, are summarized in Table 1. In the remainder of this paper, the selected conditions are named according to the nomenclature provided in Table 1.

As previously mentioned, the largest deviations in the thermal expansion behavior, when compared to the counterpart specimens, can be seen for the P 1 condition (cf. Figure 2). In contrast, all other parameter combinations selected are characterized by a plateau between 50 °C and 200 °C, which can clearly be attributed to the Invar effect explained earlier. Above T_C_ at approximately 280 °C, the thermal expansion of the specimen changes to a conventional linear slope, since the magnetic properties and, thus, the driving force of the Invar effect disappear [29]. In contrast, Condition P 1 shows virtually no plateau and, thus, no Invar effect at all. Instead, this specimen reveals an almost linear thermal expansion, which would be expected in conventional structural material. However, as can be derived from Figure 2, the CTE of this parameter set is characterized by negative values right at the beginning of the temperature range considered. As within the DOE presented in this study further parameter combinations with both similar volume energies and densities compared to Condition P 1 are available (these clearly showing the Invar effect), an explanation for the exceptional CTE behavior of Parameter Set 1 cannot be derived from the data available up to this point.

**Figure 3:**
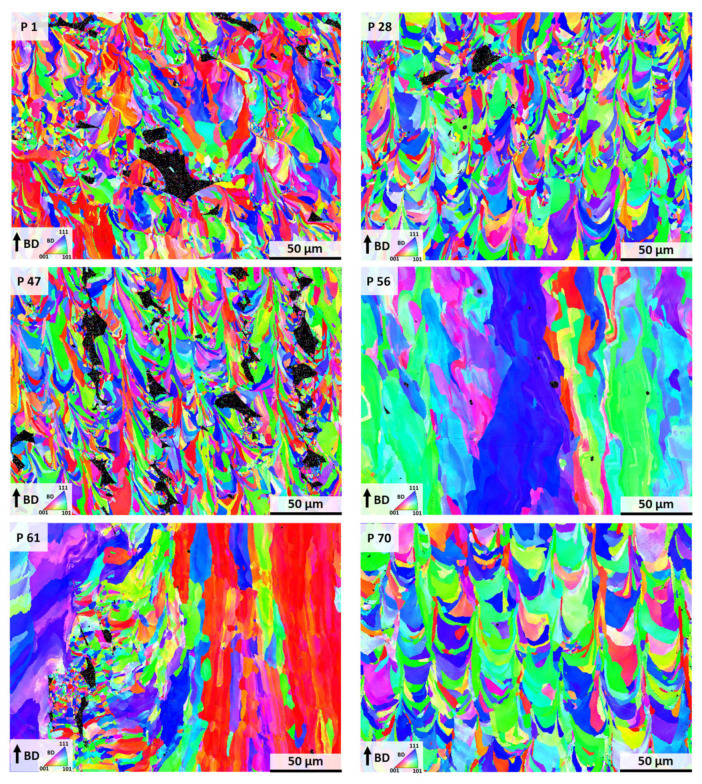
EBSD inverse pole figure (IPF) maps of PBF-LB/M-processed Invar for different selected parameter sets (see text for details). The grain orientations are plotted with respect to the build direction indicated by the arrow in the lower left marked with BD.

In order to elaborate a rationale for the differences in the thermal expansion properties, microstructural analysis by EBSD was conducted. Figure 3 shows the representative EBSD inverse pole figure (IPF) maps of the selected PBF-LB/M-processed Invar conditions. Depending on the parameter combination, a strongly different microstructure is formed with respect to porosity, as well as grain morphology and preferred orientation. The conditions P 1, P 28, P 47, and 70 show a sickle-shaped, fan-like grain structure following the dimension of the melt pools. Similar microstructures have already been reported in numerous studies on PBF-LB/M-processed Invar [16,22,30]. In contrast, the microstructures from Conditions P 56 and P 61 exhibit a columnar appearance characterized by grains having widths of 50 µm and lengths of up to 200 µm. In accordance, these two conditions are also characterized by the largest average grain sizes as determined from the EBSD measurements (cf. Supplementary Data Table S3). These two specimens were manufactured with the highest volume energies of the six selected parameter combinations considered here. Accordingly, a higher volume energy combined with a high laser power promotes the evolution of a columnar microstructure. This type of microstructure is common in many AM-processed cubic alloys showing no phase transformation upon solidification and cooling and has numerously been reported in the literature, e.g., in [31,32,33]. The shape of this microstructure is based on solidification alongside the direction of heat flow. However, comparing the CTE behavior of all selected conditions, despite P 1, as displayed in Figure 2, the partially significant differences in the grain sizes do not appear to have any pronounced influence on the CTE behavior. The same holds true for the phase compositions of the conditions in focus. The specimens of all selected parameter sets are characterized by a fully face-centered cubic (fcc) austenitic microstructure (cf. Supplementary Data Table S3) as determined from the EBSD data.

For a deeper understanding of the observations detailed, it is necessary to further assess the structure of pores and the defect types for the six parameter sets in focus. These are summarized in Table S3 of the Supplementary Data. Conditions P 1, P 47, and P 56 with densities below 97.5% show multiple defects indicating insufficient process parameters with respect to density. Here, the most remarkable defect structure can be observed for Condition P 47 (Figure 3 and Figure 5). These stripe-like pores have also been reported by Engelhardt et al. [34] in studies reporting on the variation and modeling of process parameters in PBF-LB/M-processed AlSi10Mg. The authors attributed the formation of these stripe pores to an insufficient overlap of the individual melt pools, eventually leading to an insufficient bonding and, thus, to this regular arrangement of defects. In contrast, Conditions P 1 and P 28 primarily show the appearance of a pronounced lack of fusion defects, which clearly can be seen on the fracture surfaces (cf. Figure 5), which will be discussed in more detail in the following paragraph. Lack of fusion defects are resulting from an insufficient level of energy, which can be caused by multiple factors, e.g., a too low volume energy, leading to unmolten powder particles and poor bonding [35]. This type of process-induced defect also becomes obvious for Condition P 61. However, in contrast to P 1 and P 28, the defects are not as pronounced. The density-lowering defects in the condition P 56 and, in particular, in P 70 are mainly characterized by a relatively high sphericity. Accordingly, these can either be attributed to hollow powder particles stemming from the gas-atomized pre-curser powder or keyholing, i.e., immoderate high energy leading to collapsed melt pools, which entrap gas that cannot escape from the melt pool due to the rapid solidification during PBF-LB/M [36,37]. Interestingly, Parameter 70 processed with a volume energy of 30 J/mm^3^ resulted in the most dense specimen despite not being processed within the range of volume energy recommended by Yakout et al. [6,17]. As a result, it can be concluded that the density of PBF-LB/M-manufactured components is not exclusively determined by the volume energy, as rather a large variety of parameters influences the resulting density of the built part. Overall, a closer look at the CTEs displayed in Figure 2 clearly reveals that a correlation between the defect type and the thermal expansion behavior is not possible, as similar properties can be seen for conditions revealing either lack of fusion defects or keyholing as a main defect type, as well as almost dense specimens. Since the microstructure (grain size, grain morphology, defect type) does not seem to have any influence on the CTE behavior, residual stress states, as e.g., assumed in [23] without experimental evidence, should be considered as another important factor. However, analysis of residual stress is far beyond the scope of the present study, and thus will be the subject of future work.

### 3.3. Behavior under Quasi-Static Loading

**Figure 4:**
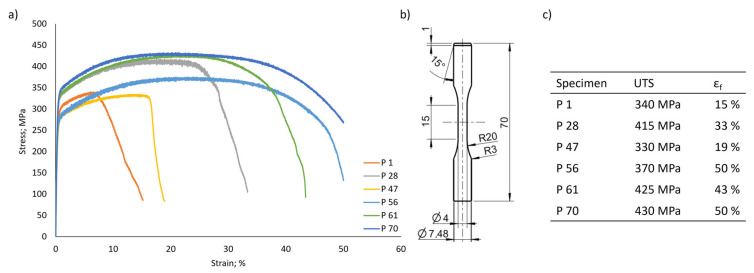
(**a**) Tensile stress-strain diagrams of representative PBF-LB/M Invar specimens processed with different parameter sets; (**b**) tensile specimen geometry employed for mechanical tests (dimensions in mm); the table in (**c**) summarizes the UTS and elongation at fracture values obtained for the parameter sets considered.

As many of the envisaged applications for Invar are used under complex loading scenarios, not only superior dimensional stabilities as a function of the temperature, but also the mechanical properties are of high interest. Therefore, in addition to the hardness measurements discussed above (detailed results are provided in Table S4 of the Supplementary Data), the six previously selected PBF-LB/M Invar specimens featuring different densities, volume energies, CTE values and resulting microstructures were characterized by tensile testing. The behavior under monotonic tensile loading is illustrated in Figure 4a by representative stress-strain curves obtained for all conditions considered.

Comparing the tensile behavior of the six parameter conditions considered, the most significant differences become apparent when considering the elongation at fracture. These deviations in the ductility can be rationalized by the densities of the different specimens. Condition P 1 (86.37% relative density) shows the lowest elongation at fracture with approximately 15%, followed by P 47 (91.58% relative density), P 28 (98.02% relative density), P 61 (97.7% relative density), P 56 (97.25% relative density), and P 70 (98.51% relative density). As already reported for various materials [22,38,39], the elongation at fracture generally increases with increasing density as the effective cross-section of the specimen is reduced by any significant amount of porosity. If the elongation at fracture is comparatively high, the PBF-LB/M Invar specimens generally reach UTS values of up to 430 MPa, as demonstrated by the conditions P 61 and P 70. However, Condition P 56 deviates from this observation as the stress-strain behavior only reveals a UTS value of 370 MPa at an elongation at a break of up to 50%. One possible explanation for the differences in strength of P 56 could be the strongly pronounced columnar microstructure as shown by EBSD (cf. Figure 3). The EBSD analysis from Specimen P 56 reveals a strongly reduced number of grain boundaries perpendicular to the direction of the load. According to the Hall-Petch relationship, this means that only a few dislocations can accumulate at the grain boundaries. Thus, the strength is significantly reduced compared to the fine-grained counterpart specimens (e.g., condition P 70) [40]. Condition P 61 is also characterized by areas revealing a strongly pronounced columnar microstructure (cf. Figure 3). In contrast to Specimen P 56, the microstructure of Condition P 61 moreover consists of fine-grained areas. These fine-grained areas appear to primarily form in the direct vicinity of larger, sharp-edged lack of fusion defects. It can therefore be concluded that these defects seem to prevent the columnar growth and, thus, lead to a much finer microstructure in direct vicinity of the defects. As both conditions are characterized by an almost similar relative density, the pore structure has a significant influence. As specimen P 56 primarily shows a few small, round-shaped pores without any fine-grained areas in the direct vicinity, only a smaller number of relatively large, sharp-edged lack of fusion defects, eventually preventing the columnar grain growth as explained above, are observed in specimen P 61. In summary, the heterogeneous, partly finer microstructure of Specimen P 61 is thought to provide an explanation for the significantly increased strength compared to Specimen P 56 according to the Hall–Petch effect despite similar densities of both.

Another important aspect that needs to be considered in the context of the mechanical properties obtained by quasi-static tensile loading is the surface state of the specimen in focus. As already detailed in Section 2, the specimen of the six selected parameter sets were analyzed in an as-built condition with respect to their surface state. As a result, all specimens were characterized by adhering powder particles on the surfaces, representing a well-known phenomenon in the powder-bed-based AM [36]. However, a comparison of the surface roughness as determined by optical focus variation (cf. Table S3 in the Supplementary Data) shows that no pronounced differences can be identified for the conditions in focus being able to explain the differences in stress-strain behavior, as shown in Figure 4. Thus, the influence of the surface roughness on the mechanical properties upon tensile testing can be excluded.

In order to compare the densities resulting from the parameter sets used within the present work with the available literature on PBF-LB/M-processed Invar, several studies need to be discussed. First of all, Yakout et al. [6], who could fabricate Invar36 with a relative density of around 99% with a volume energy in the range between 60 and 75 J/mm^3^. Wei et al. [41] processed Invar36 with a volume energy of 99.2 J/mm^3^ and were able to realize a relative density of 99.5%. Moreover, Wegener et al. [16] processed Invar36 with a volume energy of 70.6 J/mm^3^, reaching a relative density of 99.6% as determined by computed tomography. Within the present study, the relative densities of the six parameter sets selected were in the range of 86.37% to 98.51%. Specimens were built using a large variety of volume energies between 27 and 133 J/mm^3^ (cf. Table 1). Within the literature, the relative density of the processed Invar parts often is directly connected to the volume energy. However, the conditions P 47, P 61, and P 70 of the present study show a clear mismatch with respect to such a conclusion. While Condition P 47 (27 J/mm^3^) shows a relative density of only 91.58%, Condition P 70 reached a relative density of 98.51%, with only a slightly higher volume energy of 30 J/mm^3^. In contrast, Parameter P 1 was processed with 57 J/mm^3^ and, therefore, almost within the range of volume energy between 60 and 75 J/mm^3^, as recommended by Yakout et al. [6,17]. However, the latter is the least-dense specimen, revealing a density of only 86.37%. Comparing the relative density of the parameter sets to the elongation at fracture as shown in Figure 4a, and Figure 4c, even parameters with a low relative density (P 1 and P 47) reveal relatively high elongation under monotonic tensile load. Clearly, comparing the results of tensile tests conducted in the present work with the literature, a high damage tolerance of the PBF-LB/M-processed Invar, as already reported by Wegener et al. [16], can be concluded. In order to analyze this damage tolerance under more critical loading scenarios, investigations on the cyclic properties of the selected parameter sets will be the subject of future studies. The pronounced damage tolerance is particularly illustrated by Condition P 70. Revealing a relative density of only 98.51%, the specimen built with this parameter is characterized by a UTS and elongation at fracture of 430 MPa and 50%, respectively. These characteristic values are in total agreement with the results presented in the literature for dense components. Wegener et al. [16] reported on specimens characterized by a relative density of 99.6%, which revealed a UTS and elongation at fracture of 445 MPa and 47%, respectively. Similar values were also reported by Qui et al. [22]. The vertically built and heat-treated specimens in that study were characterized by an average UTS of approximately 445 MPa as well as by elongations at fracture of about 30%. In addition, UTS values of about 450 MPa with a concomitant ductility of 50% were reported by Yakout et al. [20] for specimens featuring the highest density.

**Figure 5:**
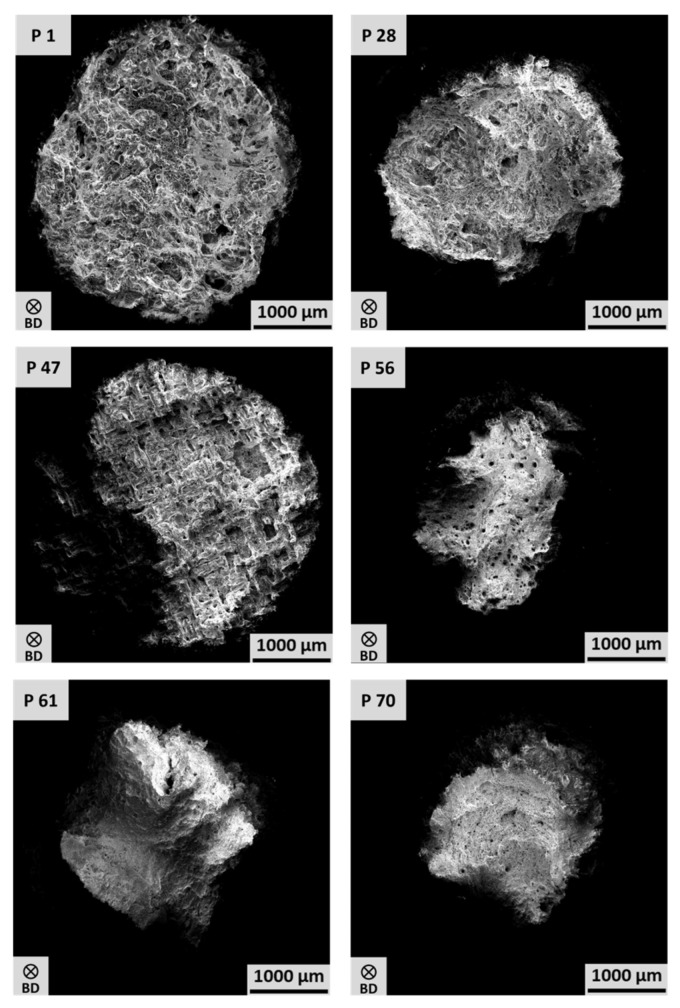
SEM micrographs of representative fracture surfaces for the parameter sets investigated in the tensile tests.

The results of the fracture surface analysis in Figure 5 confirm the correlation between relative density and elongation at fracture elaborated from the tensile tests. The conditions P 56, P 61, and P 70 are characterized by pronounced necking and a high degree of deformation, respectively, leading to the well-known cup and cone fracture-surface appearance, as already shown by Wegener et al. [16]. This appearance can be attributed to the ductile fracture behavior, with the high elongation at fracture (cf. Figure 4) being characteristic of these parameter sets. Comparing the fracture surfaces of the conditions P 56 and P 70, interesting differences can be deduced. Although both conditions show a similar elongation at fracture of about 50%, the appearance of the fracture surface is different. While Specimen P 70 shows uniform necking, Specimen P 56 is characterized by a non-uniform necking. This observation could be attributed to the strongly columnar microstructure consisting of only several individual grains within the gauge length of Specimen P 56. Due to the significantly reduced number of grains compared to Specimen P 70, the local deformation is likely guided by the larger grains. Eventually, this leads to inhomogeneous necking, whereas the relatively homogeneous fine-grained microstructure of Specimen P 70 results in a more uniform deformation, leading to almost homogeneous necking in a circular shape. These considerations are further supported by the fracture surface appearance of Specimen P 61, as partly inhomogeneous necking can be likewise observed. However, compared to Condition P 56, this observation is not as pronounced, eventually being in line with the more or less bimodal microstructure not only consisting of columnar grains (cf. Figure 3). From the results of the fracture surface analysis presented, the different previously described defects (cf. also Table S3 of the Supplementary Data) deduced from the EBSD data in Figure 3 are also noticeable. The fracture surfaces of Specimens P 1 and P 28 reveal pronounced and large lack of fusion defects, which can be recognized by the characteristic, unmelted powder particles. In line with the results from microstructural analysis, these defects are not as pronounced on the fracture surface of Condition P 61. A special characteristic is obvious in the case of the fracture surface of Condition P 47, in which the pores are regularly distributed in the order of the distance between the hatch lines. As discussed before, such a regular arrangement of stripe pores was also found by Engelhardt et al. [34], attributing the formation of these defects to an insufficient overlap of the individual narrow pools, leading to an insufficient bonding and, thus, to this regular arrangement of defects. In contrast, mostly round-shaped defects with relatively high sphericity, probably indicating keyholes, can be deduced from the fracture surface of Condition P 56.

## 4. Summary and Conclusions

In the present study, a full-factorial experimental design for the processing of Invar using PBF-LB/M was considered. Here, the processability could be demonstrated over a large parameter range, eventually allowing a specific design of the microstructure and relative density. The main aspects can be summarized as follows:

1. In contrast to the statements provided in literature, no generally valid positive effect of increased porosity on thermal expansion could be revealed in the present study (considering by far the largest processing window in the literature). Both, high and low relative density parameter combinations are characterized by low thermal expansion. This is also underlined by statistical analysis of the DOE as Pearson’s correlation coefficients close to zero are reported for a correlation between CTE and density. Most importantly, many parameter combinations show the characteristic CTE of Invar within the temperature range from 50–200 °C.
2. The microstructure being established by the different parameter combinations differs significantly. For example, high volume energy results in a coarse-grained columnar structure, whereas lower volume energies lead to a sickle-shaped fan-like grain structure following the size of the melt pools.
3. Depending on the volume energy of the parameter sets used during PBF-LB/M, the microstructures of the conditions investigated reveal different defect types such as lack of fusion or keyhole porosity. A correlation between defect type and thermal expansion behavior could not be found.
4. The different relative densities and microstructures also affect the mechanical behavior, as revealed by tensile testing. Low-density specimens show significantly reduced elongation at fracture, whereas high densities result in elongations at fractures of up to 50%. A coarse-grained columnar microstructure with few grain boundaries perpendicular to the loading direction presumably leads to reduced ultimate tensile strength.
5. The results from the tensile tests are confirmed by fracture-surface analysis. Specimens with high elongation at fracture are characterized by pronounced necking, leading to a well-known cup and cone fracture shape.
6. The results of the present investigation confirm the high potential of Invar to exhibit low-thermal expansion over a wide range of processing parameters. In addition, it is possible to specifically adjust the microstructure in order to adapt the mechanical properties to envisaged applications.

## Figures and Tables

**Table 1:** Summary of the process parameters (laser power p, scanning speed s, hatch h, and layer thickness t), as well as volume energy E_V_ and the density of the parameter combinations selected for microstructural analysis and tensile testing.

DOE Number	Nomenclature	P in W	v_s_ in mm/s	h in mm	t in mm	E_V_ in J/mm^3^	Density in %
1	P 1	100	700	0.05	0.05	57	86.37
28	P 28	200	700	0.1	0.1	29	98.02
47	P 47	200	1000	0.15	0.05	27	91.58
56	P 56	300	800	0.05	0.1	75	97.25
61	P 61	300	900	0.05	0.05	133	97.7
70	P 70	300	1000	0.1	0.1	30	98.51

## Data Availability

The raw data supporting the conclusions of this article will be made available by the authors upon request.

## Supplementary Materials

The following supporting information can be downloaded at: https://www.mdpi.com/article/10.3390/ma17174313/s1, Figure S1: Analysis of the DOE with statistical methods using the software Minitab^®^: Pearson correlation analysis for (a) the CTE in the temperature range 50–100 °C (CTE 1) and density and (b) the CTE in the temperature range 100–200 °C (CTE 2) and density; Figure S2: Analysis of the DOE with statistical methods using the software Minitab^®^: Results of the influence analysis (DOE). Evaluation of all investigated influence parameters of the full factorial investigation for (a) CTE1 and (b) CTE2; Table S1: Design of Experiments used for manufacturing of Invar via PBF-LB/M. The parameter sets used for the following investigation are marked in bold letters; Table S2: First results from the Design of Experiments including relative density, hardness, and thermal expansion coefficient (αt_h_). The parameter sets used for the following investigation are marked in bold letters; Table S3: Summary of various microstructural features of the six selected parameter sets for microstructural analysis and quasi-static tensile testing; Table S4: Summary of the hardness measurements for the six selected parameter sets for microstructural analysis and quasi-static tensile testing.
