# Supplementary material for: Influence of Defects and Microstructure on the Thermal Expansion Behavior and the Mechanical Properties of Additively Manufactured Fe-36Ni
Source: Materials (Basel). 2024 Aug 30;17(17):4313. doi: 10.3390/ma17174313 (PMC11395997; doi:10.3390/ma17174313)
Supplement: Supplementary file 1 [file materials-17-04313-s001.zip › materials-3126546-supplementary.pdf]

Supplementary Material

Table S1. Design of Experiments used for manufacturing of Invar via PBF-LB/M. The parameter sets used for the following investigation are marked in bold letters.

Number	Power; W	velocity; mm/s	hatch; mm	thickness; mm	Volume energy; J/mm³
**1**	**100**	**700**	**0.05**	**0.05**	**57**
2	100	700	0.05	0.1	29
3	100	700	0.1	0.05	29
4	100	700	0.1	0.1	14
5	100	700	0.15	0.05	19
6	100	700	0.15	0.1	10
7	100	800	0.05	0.05	50
8	100	800	0.05	0.1	25
9	100	800	0.1	0.05	25
10	100	800	0.1	0.1	13
11	100	800	0.15	0.05	17
12	100	800	0.15	0.1	8
13	100	900	0.05	0.05	44
14	100	900	0.05	0.1	22
15	100	900	0.1	0.05	22
16	100	900	0.1	0.1	11
17	100	900	0.15	0.05	15
18	100	900	0.15	0.1	7
19	100	1000	0.05	0.05	40
20	100	1000	0.05	0.1	20
21	100	1000	0.1	0.05	20
22	100	1000	0.1	0.1	10
23	100	1000	0.15	0.05	13
24	100	1000	0.15	0.1	7
25	200	700	0.05	0.05	114
26	200	700	0.05	0.1	57
27	200	700	0.1	0.05	57

**28**	**200**	**700**	**0.1**	**0.1**	**29**
29	200	700	0.15	0.05	38
30	200	700	0.15	0.1	19
31	200	800	0.05	0.05	100
32	200	800	0.05	0.1	50
33	200	800	0.1	0.05	50
34	200	800	0.1	0.1	25
35	200	800	0.15	0.05	33
36	200	800	0.15	0.1	17
37	200	900	0.05	0.05	89
38	200	900	0.05	0.1	44
39	200	900	0.1	0.05	44
40	200	900	0.1	0.1	22
41	200	900	0.15	0.05	30
42	200	900	0.15	0.1	15
43	200	1000	0.05	0.05	80
44	200	1000	0.05	0.1	40
45	200	1000	0.1	0.05	40
46	200	1000	0.1	0.1	20
**47**	**200**	**1000**	**0.15**	**0.05**	**27**
48	200	1000	0.15	0.1	13
49	300	700	0.05	0.05	171
50	300	700	0.05	0.1	86
51	300	700	0.1	0.05	86
52	300	700	0.1	0.1	43
53	300	700	0.15	0.05	57
54	300	700	0.15	0.1	29
55	300	800	0.05	0.05	150
**56**	**300**	**800**	**0.05**	**0.1**	**75**
57	300	800	0.1	0.05	75
58	300	800	0.1	0.1	38

59	300	800	0.15	0.05	50
60	300	800	0.15	0.1	25
**61**	**300**	**900**	**0.05**	**0.05**	**133**
62	300	900	0.05	0.1	67
63	300	900	0.1	0.05	67
64	300	900	0.1	0.1	33
65	300	900	0.15	0.05	44
66	300	900	0.15	0.1	22
67	300	1000	0.05	0.05	120
68	300	1000	0.05	0.1	60
69	300	1000	0.1	0.05	60
**70**	**300**	**1000**	**0.1**	**0.1**	**30**
71	300	1000	0.15	0.05	40
72	300	1000	0.15	0.1	20

Table S2. First results from the Design of Experiments including relative density, hardness, and thermal expansion coefficient (α_{th}). The parameter sets used for the following investigation are marked in bold letters.

Number	Density	Hardness	α_{th} (50-100°C)	α_{th} (100-200°C)
		HV1	10^{-6} 1/K	10^{-6} 1/K
**1**	**86.36%**	**147**	**-3.2**	**-1**
2	71.17%	116	-2.2	-0.8
3	78.49%	138	-2.2	-0.5
4	61.19%	85	-1	0.2
5	67.55%	74	-4.5	-5
6	47.41%	84	-0.4	0.6
7	88.66%	144	-1.5	0
8	71.09%	121	-0.5	0
9	78.86%	115	-1.5	-0.5
10	60.87%	102	0	0.9
11	56.56%	87	-0.2	0.8
12	38.33%	50	-0.6	0.4

13	84.78%	138	-0.5	0.3
14	71.79%	130	-1.5	0
15	69.69%	108	0.7	1
16	55.75%	79	-0.8	0.9
17	58.92%	96	-3	-0.5
18	38.59%	60	-0.4	0.5
19	88.78%	144	0	0.75
20	69.62%	111	0	0.9
21	71.48%	91	-3	-0.5
22	55.55%	89	-0.15	0.65
23	56.21%	72	-2	-0.5
24	33.00%	68	0	0.9
25	98.45%	139	-0.5	0.5
26	98.01%	142	0.8	1
27	98.61%	144	-2	-0.5
**28**	**98.02%**	**143**	**1.2**	**1.4**
29	98.73%	139	-2	-0.5
30	88.14%	136	0.7	0.8
31	97.97%	136	0.3	1
32	98.70%	139	0	0.8
33	99.67%	148	-4	-1.5
34	99.64%	143	0.3	1.1
35	99.31%	128	0.5	1
36	88.69%	123	-3	-1
37	99.25%	147	-3	-1
38	98.58%	147	-0.5	0.5
39	99.38%	148	0.5	1
40	97.61%	147	-2	-0.7
41	93.65%	134	-0.5	0.7
42	70.77%	121	0	0.4
43	98.82%	150	-0.4	0.4

44	95.69%	148	-0.8	0.5
45	99.31%	153	-2	-0.6
46	86.69%	135	0	0.9
**47**	**91.58%**	**135**	**0**	**0.5**
48	72.88%	118	0.85	0.8
49	97.93%	145	0	0.7
50	97.71%	144	0.9	1.15
51	98.89%	149	-1.7	-0.3
52	98.32%	141	0.2	0.6
53	99.66%	150	-1	-0.2
54	98.42%	141	0.4	0.65
55	98.96%	144	0.5	0.8
**56**	**97.25%**	**136**	**0.3**	**0.5**
57	96.10%	144	-1	0.3
58	98.26%	142	-0.5	0.6
59	98.36%	149	-2.3	-0.6
60	98.23%	136	0.6	1
**61**	**97.70%**	**138**	**0.2**	**0.5**
62	98.21%	140	0.3	1
63	97.37%	148	-2.5	-0.6
64	98.85%	148	0.2	0.6
65	98.65%	150	0.5	0.8
66	96.40%	122	0	0.3
67	98.66%	139	-0.4	0.5
68	96.33%	142	-0.5	0.5
69	97.21%	144	-3	-1
**70**	**98.51%**	**142**	**-0.6**	**0.2**
71	97.58%	144	0.3	0.7
72	91.99%	136	-3	-1

Table S3. Summary of various microstructural features of the six selected parameter sets for microstructural analysis and quasi-static tensile testing.

Specimen	Phase composition	Grain size, µm	Main defect type	Surface roughness, µm
P 1	fcc	34.4	Lack of fusion	101.9
P 28	fcc	36.3	Lack of fusion	121.4
P 47	fcc	30.2	Stripe pores	101.5
P 56	fcc	220	Keyholes	152.4
P 61	fcc	110	Lack of fusion	91.9
P 70	fcc	59.4	Mostly dense	82.6

Table S4. Summary of the hardness measurements for the six selected parameter sets for microstructural analysis and quasi-static tensile testing.

Specimen	Diagonal 1, mm	Diagonal 2, mm	Hardness, HV1	Average hardness, HV1
P 1	0.1121	0.11	150	
	0.1144	0.112	145	147
	0.1177	0.1068	147	
P 28	0.1132	0.1139	144	
	0.1138	0.1134	144	143
	0.1151	0.1132	142	
P 47	0.1144	0.1122	144	
	0.117	0.112	142	135
	0.1259	0.1234	119	
P 56	0.1186	0.1181	132	
	0.1156	0.1154	139	136
	0.1166	0.1161	137	
P 61	0.1165	0.1142	139	
	0.1163	0.1156	139	138
	0.1156	0.1173	136	
P 70	0.1156	0.1161	138	
	0.1137	0.1117	146	142
	0.1151	0.1129	143	

a)

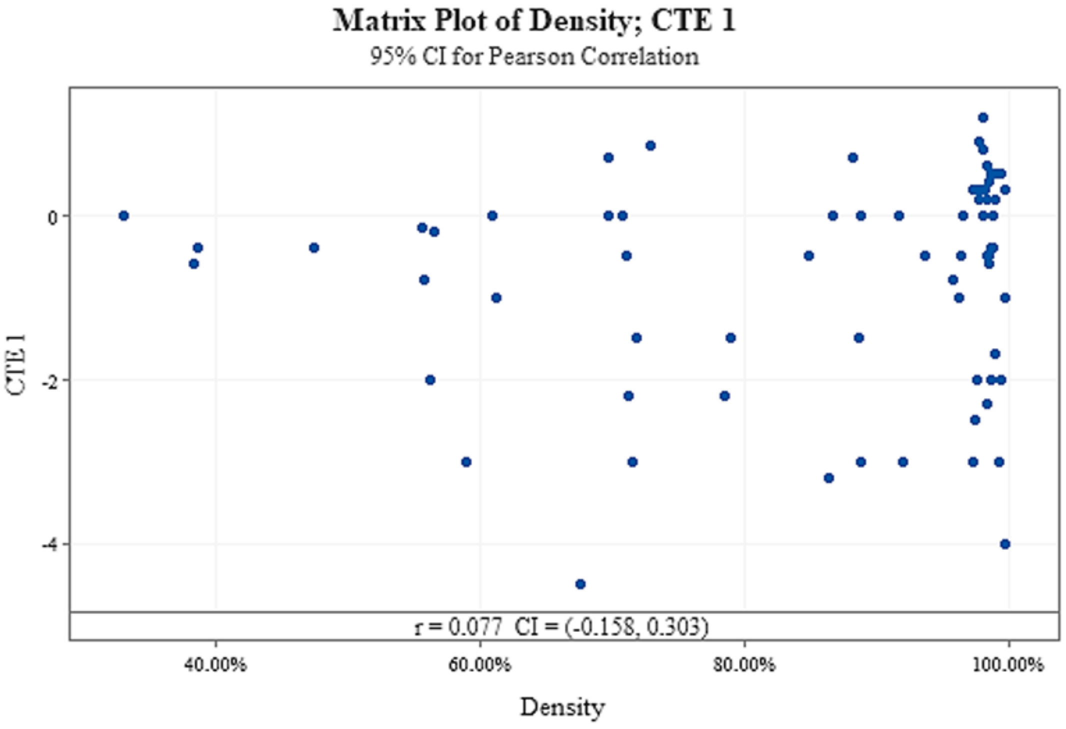

b)

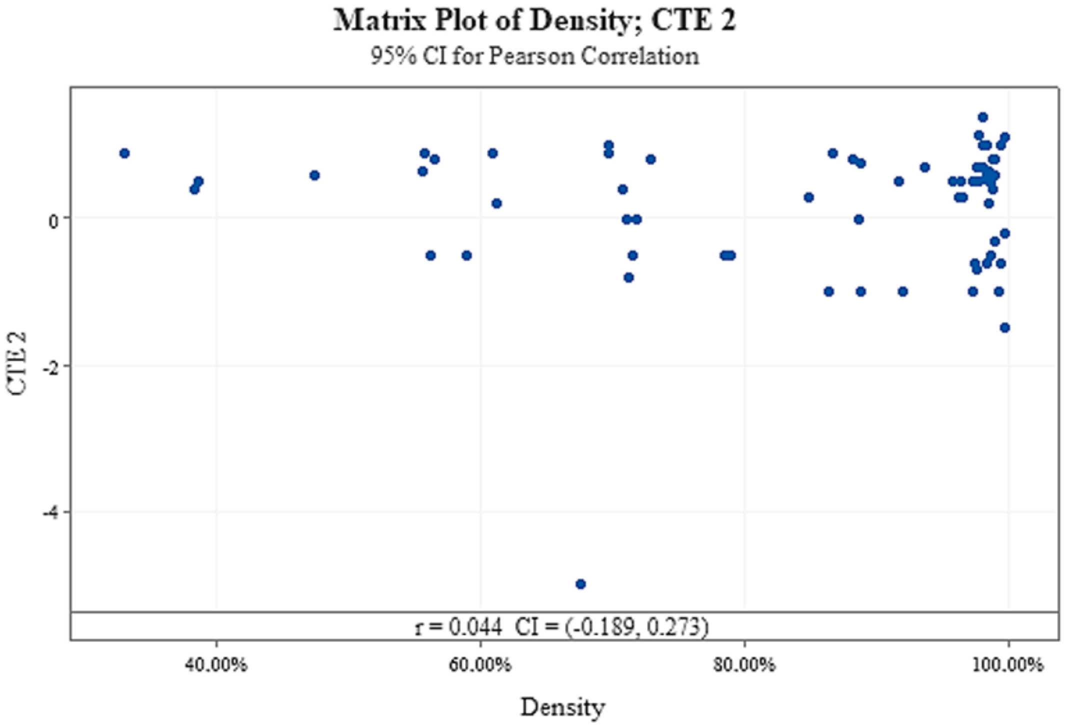

Figure S1. Analysis of the DOE with statistical methods using the software Minitab®: Pearson correlation analysis for a) the CTE in the temperature range 50-100 °C (CTE 1) and density and b) the CTE in the temperature range 100-200 °C (CTE 2) and density.

a)

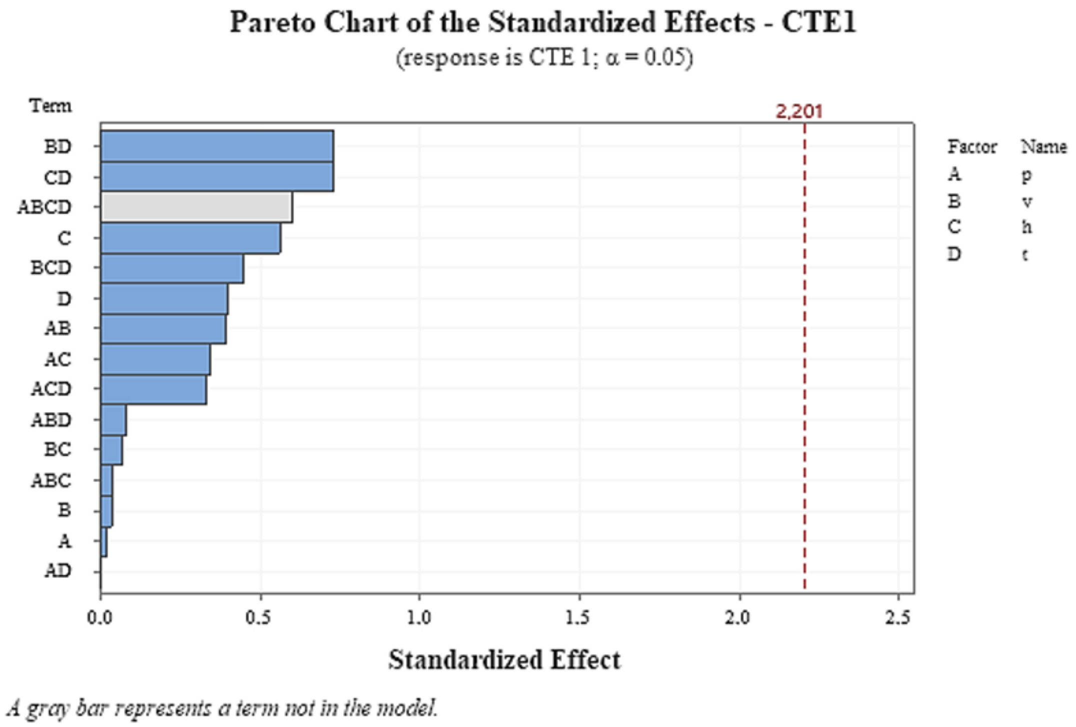

b)

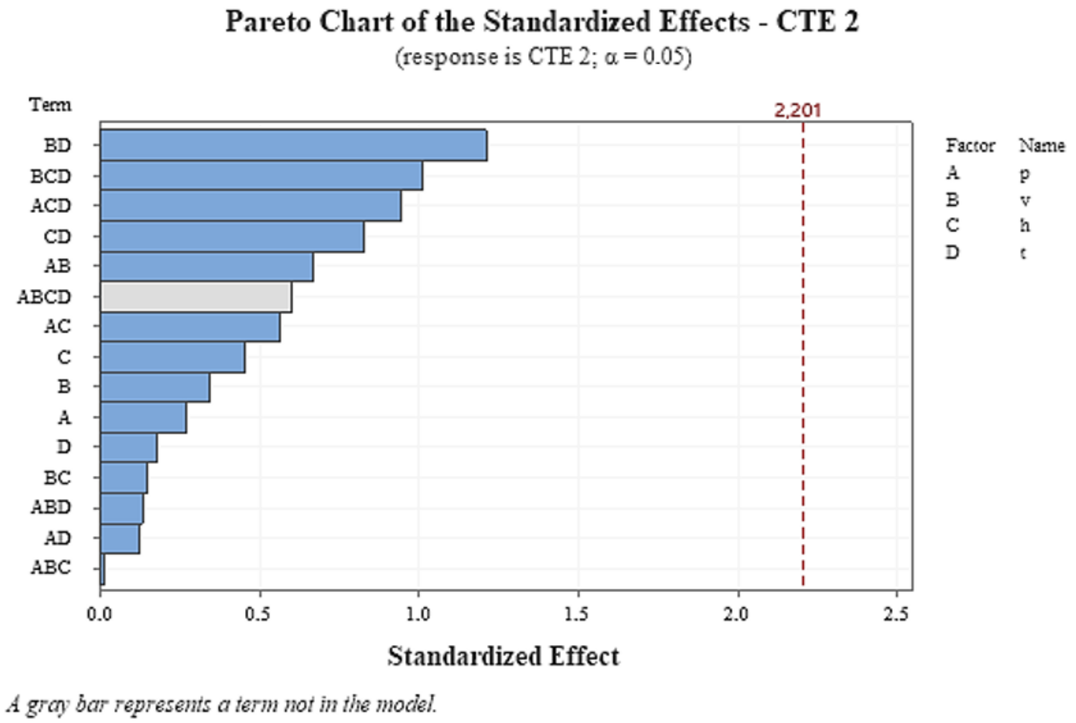

Figure S2. Analysis of the DOE with statistical methods using the software Minitab®: Results of the influence analysis (DOE). Evaluation of all investigated influence parameters of the full factorial investigation for a) CTE1 and b) CTE2.
